# Hypoalbuminaemia in haemodialysis patients at Parirenyatwa group of hospitals and Chitungwiza central hospital

**DOI:** 10.11604/pamj.2015.21.79.4171

**Published:** 2015-06-01

**Authors:** Pasipanodya Ian Machingura, Needmore Muchadura Mahiya, Vasco Chikwasha

**Affiliations:** 1 University of Zimbabwe College of Health Sciences Department of Medical Laboratory Sciences, Zimbabwe; 2 University of Zimbabwe College of Health, Sciences Department of Community Medicine, Zimbabwe

**Keywords:** Hypoalbuminaemia, haemodialysis, Parirenyatwa, Chitungwiza

## Abstract

**Introduction:**

Haemodialysis is one of the widely used methods in end stage renal disease. However it has a negative impact on the quality of life of the renal patients. Hypoalbuminaemia occur in haemodialysis patients and it correlates strongly with mortality and morbidity. We sought out to determine the prevalence of hypoalbuminaemia among haemodialysis patients at Parirenyatwa group of hospitals and Chitungwiza central hospital.

**Methods:**

A questionnaire was administered on haemodialysis patients at Parirenyatwa Group of Hospitals and Chitungwiza Central Hospital who consented to participate in the study. Pre dialysis serum samples collected from the patients were used for albumin analysis. The serum from the patients was analysed for serum albumin levels using the Mindray BS120 chemistry analyser using the bromocresol green method.

**Results:**

A total of 60 patients were recruited from the two hospitals. The Mean albumin concentration for the entire group was 33.6g/L SD (6.1 g/L). The mean albumin in males was 33.6 g/L, SD (5.9) and in female 33.6, SD (6.6 g/L) and this was not statistically significantly different (p = 0.988). The prevalence of hypoalbuminaemia was 76.7%.

**Conclusion:**

Hypoalbuminaemia in 76.7% of haemodialysis patients studied is a cause of concern thus monitoring of haemodialysis patients albumin is necessary since its decreased levels has been associated with increased morbidity and mortality.

## Introduction

End stage renal disease is an important public health problem. Renal replacement therapy, dialysis and transplantation are used to provide relief from the symptoms of end stage renal disease whilst also preserving the life of the patients though they are not curative. Haemodialysis is the widely used method although it produces a negative impact on quality of life of the patients [1]. Hypoalbumianemia has been reported to be frequently present in haemodialysis patients and correlates strongly with mortality and morbidity. Levels of albumin are decreased in patients with inflammatory states, thus it's not clear to what extent hypoalbuminemia is due to malnutrition or simply a reflection of inflammatory states [2]. Patients with advanced chronic kidney disease who receive chronic hemodialysis treatment suffer from various degrees of protein energy wasting. Low serum albumin is one of the key diagnostic criteria proposed by the International Society of Renal Nutrition Experts Panel [3]. Serum albumin is still the most common nutritional marker used in end stage renal disease patients [4]. Serum albumin measurement is a simple and readily available test [5]. We sought out to determine the prevalence of hypoalbuminaemia among haemodialysis patients at Parirenyatwa group of hospitals and Chitungwiza central hospital.

## Methods

### Data and sample collection

Permission to carry out the project was sought from the authorities at Parirenyatwa Group of Hospitals, Chitungwiza Central Hospital and Joint Research Ethics Committee of the College of Health Sciences. Only patients who consented were recruited into the study between January and February 2013. A questionnaire was administered on haemodialysis patients at Parirenyatwa Group of Hospitals and Chitungwiza Central Hospital who consented to participate in the study. The questionnaire was used to collect demographic information and other information from the patient. Serum from the pre dialysis blood samples collected from the patients was used for albumin analysis.

### Biochemical analysis

The serum from the patients was analysed for serum albumin levels using the Mindray BS120 chemistry analyser using the bromocresol green method. The analyser was calibrated and control samples were analysed before analysis of samples. Samples were only analysed when the control samples were within their reference range. The samples were processed and results were recorded for statistical analysis.

### Data analysis

The data was captured using Microsoft excel and exported to Stata 12 for cleaning and analysis. Patients characteristics were summarized using mean (SD) for continuous normally distributed variables. Categorical variables were summarized using frequencies and percentages. Hypoalbuminaemia was defined as serum albumin less than 38g/l [3, 5, 6].

## Results

A total of 60 patients were enrolled into the study with a mean age of 46.7 years, SD (13.5 years). The majority of the patients 70% (42/60) were male. There was no significant difference in age distribution between males (mean age = 48.5 years, SD = 12.8 years) and females (mean age = 42.7 years, SD = 14.6 years), (p = 0.134). Mean albumin concentration for the entire group was 33.6g/L SD (6.1 g/L). The mean albumin in males was 33.6 g/L, SD (5.9) and in female 33.6, SD (6.6 g/L) and this was not statistically significantly different (p = 0.988). Prevalence of hypoalbuminaemia was 76.7% (Table 1). Being female had a protective effect against hypoalbuminaemia compared to being male but this was not statistically significant, OR = 0.71, 95% CI (0.20-2.52), p = 0.595. The odds of developing hypoalbuminaemia increased with age but was not statistically significant, OR = 1.02, 95% CI (0.98-1.07), p = 0.318. Period on dialysis was not a significant predictor of hypoalbuminaemia OR = 0.99, 95% CI (0.98-1.01). Having BP was not a significant predictor of hypoalbuminaemia, OR = 1.75, 95% CI (0.29-10.74), p = 0.546.

**Table 1:** Prevalence of hypoalbuminaemia

Albumin category	Frequency	Percent Frequency
Normal	14	23.3
hypoalbuminaemia	46	76.7

## Discussion

It has been reported that morbidity and mortality incidence of dialysed patients with hypoalbuminemia is higher than in patients with normal serum albumin [7]. Mean albumin concentration for the entire group was 33.6 g/L SD (6.1 g/L). The mean albumin in males was 33.6 g/L, SD (5.9) and in female 33.6, SD (6.6 g/L) and this was not statistically significantly different (p = 0.988). This is a cause of concern since patients with a serum albumin levels below 35g/L have been reported to have a relative mortality risk of 4 or a 2 year survival of 20% when compared with a 2 year survival of 80% in those with serum albumin greater than 40g/L [8]. The mean albumin of the entire group was less than 40.7g/L reported in 300 end stage renal disease patients in Tehran University of Medical Sciences Hospital during the year 2010 [4].

In dialysis patients low serum albumin level indicates more severe comorbidity, worse general health status and protein energy wasting [3]. The prevalence of hypoalbuminemia in this study was found to be 76.7% which is lower than that reported in Pakistan of 90.6% [9]. The study in Parkistan used a cut of for hypoalbuminaemia of 40g/L which is more than the 38g/L which is currently recommended. In dialysis patients the amino acid and protein losses during the dialysis session and the low nutrient intake leads to low nutrient availability for muscle synthesis [10]. Oral nutritional supplements and other nutritional interventions to chronic kidney disease patients have been suggested to increase serum albumin levels and improve longevity and their quality of life [5]. Several studies have reported that haemodialysis patients eat less protein and fewer calories than prescribed and are associated with higher rates of malnutrition [8].

## Conclusion

Hypoalbuminaemia in 76.7% of haemodialysis patients studied is a cause of concern thus monitoring of haemodialysis patients albumin is necessary since its decreased levels has been associated with increased morbidity and mortality.
